# Maternal and Fetal Placental Growth Hormone and IGF Axis in Type 1 Diabetic Pregnancy

**DOI:** 10.1371/journal.pone.0029164

**Published:** 2012-02-17

**Authors:** Mary F. Higgins, Noirin E. Russell, Paul A. Crossey, Kristine C. Nyhan, Derek P. Brazil, Fionnuala M. McAuliffe

**Affiliations:** 1 UCD Obstetrics and Gynaecology, School of Medicine and Medical Science, University College Dublin, National Maternity Hospital, Dublin, Ireland; 2 UCD Conway Institute of Biomolecular and Biomedical Research, University College Dublin, Dublin, Ireland; 3 Centre for Vision and Vascular Science, Queen's University Belfast, Belfast, Northern Ireland, United Kingdom; Mayo Clinic College of Medicine, United States of America

## Abstract

**Aim:**

Placental growth hormone (PGH) is a major growth hormone in pregnancy and acts with Insulin Like Growth Factor I (IGF-I) and Insulin Like Growth Hormone Binding Protein 3 (IGFBP3). The aim of this study was to investigate PGH, IGF-I and IGFBP3 in non-diabetic (ND) compared to Type 1 Diabetic (T1DM) pregnancies.

**Methods:**

This is a prospective study. Maternal samples were obtained from 25 ND and 25 T1DM mothers at 36 weeks gestation. Cord blood was obtained after delivery. PGH, IGF-I and IGFBP3 were measured using ELISA.

**Results:**

There was no difference in delivery type, gender of infants or birth weight between groups. In T1DM, maternal PGH significantly correlated with ultrasound estimated fetal weight (r = 0.4, p = 0.02), birth weight (r = 0.51, p<0.05) and birth weight centile (r = 0.41, p = 0.03) PGH did not correlate with HbA1c.

Maternal IGF-I was lower in T1DM (p = 0.03). Maternal and fetal serum IGFBP3 was higher in T1DM. Maternal third trimester T1DM serum had a significant band at 16 kD on western blot, which was not present in ND.

**Conclusion:**

Maternal T1DM PGH correlated with both antenatal fetal weight and birth weight, suggesting a significant role for PGH in growth in diabetic pregnancy.

IGFBP3 is significantly increased in maternal and fetal serum in T1DM pregnancies compared to ND controls, which was explained by increased proteolysis in maternal but not fetal serum. These results suggest that the normal PGH-IGF-I-IGFBP3 axis in pregnancy is abnormal in T1DM pregnancies, which are at higher risk of macrosomia.

## Introduction

Type 1 Diabetes (T1DM) affects less than 1% of the obstetric population but is a significant cause of fetal and neonatal morbidity and mortality [1]–[3]. Many of these complications are related to fetal macrosomia [4] that, despite enhancements in glycaemic control, is still common in diabetic pregnancies [4], [5]. Macrosomia is related to an increased risk of unexplained in-utero death, instrumental delivery, shoulder dystocia and later life obesity [6]–[8].

Fetal growth is a complex process influenced by genetics, maternal factors, uterine environment and maternal and fetal hormones [4], [9]. Diabetic mothers have an additional influence of maternal hyperglycaemia or fluctuating glucose. Differences in HbA1c may explain 6–23% of differences in birth weight [7]. Additionally, the influence of growth factors, both maternal and fetal, may be of importance [4].

Human Placental Growth Hormone (PGH) which is secreted by the syncytiotrophoblast [10] Is found in the maternal circulation from the sixth week of gestation [11] and gradually replaces pituitary growth hormone during pregnancy. PGH, also known as growth hormone variant (GHV) was traditionally thought to be found only in maternal blood and to influence fetal growth by regulating maternal supply of nutrients to the fetus via the placenta [11]. The effect of PGH on fetal growth may be mediated by a direct autocrine and paracrine mechanisms or via regulation of Insulin Growth Factor 1 (IGF-I) [12]. As in non-diabetic pregnancies, there is a positive correlation between maternal PGH and birth weight in women with diabetics [12]. However, no studies to date have demonstrated higher levels of *total* serum maternal PGH in diabetic pregnancies, despite the higher incidence of macrosomia in the infants of diabetic mothers. In one study the mean level of *free* PGH were significantly higher in pregnant women with Type 1 Diabetes (T1DM), possibly associated with the fall in growth hormone binding protein [13]. In the placenta itself, the ratio of PGH to chorionic sommatotrophin L (CS-L) mRNA is raised compared to non diabetic term placenta [14]. Both suggest that increased PGH levels drive increased glucose levels and increased insulin resistance.

IGF-I is one of two growth factors in the IGF family. IGF-I has both mitogenic and anabolic actions [15]. The biological actions of the IGFs are regulated at the level of bioavailability through association with a family of specific high affinity binding proteins, termed the IGF binding proteins (IGFBPs). The ligand binding affinity of the IGFBPs can be modulated by phosphorylation and proteolysis.

IGF-I is decreased in maternal serum in diabetic pregnancies compared to non-diabetic controls [16]. Cord serum of IGF1 is increased in infants of insulin treated mothers (T1DM and T2DM) compared to non diabetic controls [17].

Insulin like growth factor 3 (IGFBP3) is the predominant maternal serum IGF binding protein at term. IGFBP3 is the most abundant binding protein in human adult serum, with a high affinity for IGF, binding up to 70–80% of total serum IFG-I and IGF-II [18]. Bound IGF is unable to cross the endothelium into tissues and therefore IGFBP/IGF complexes act as a reservoir of circulating IGF, increasing the half-life from 10 minutes to 12–15 hours [19]. Cord IGFBP3 is higher in infants of T1 and T2 diabetic mothers compared to non-diabetic controls [16].

The aim of this study was to measure the PGH-IGF-I-IGFBP3 axis in non-diabetic (ND) and T1DM pregnancy. In particular, we hypothesised that fetal PGH would differ between diabetic and control pregnancies. Additionally we aimed to map in detail the inter-relationship between maternal and fetal PGH and IGF in T1DM pregnancy.

## Results

### Study Groups

25 non-diabetic and 25 T1DM women consented to participate; matching maternal and cord samples were obtained. There were no differences between the groups regarding maternal age, parity, type of delivery or birth weight. Although there was no difference in crude birth weight measurements, women with T!DM delivered at an earlier gestational age and the birth weight centile (correcting birth weight for gestational age at delivery) was higher in the T1DM group compared to the non-diabetic group T1DM participants were also more likely to deliver an infant, requiring admission to the neonatal intensive care or with a poorer composite perinatal outcome (see Table 1).

**Table 1:** Background characteristics of participants.

	Non Diabetic n = 25	T1DM n = 25	p value
**Age (years)**	32 (22–42)	33 (26–39)	0.64
**Parity (% primiparous)**	9/25	11/25	0.53
**BMI**	23.9 (18.7–31.2)	27.3 (21.8–35)	0.01
**Mode of Delivery (%Caesarean Section)**	20/25 (80%)	21/25 (84%)	0.65
**Gender of infant (% female)**	10/25 (40%)	16/25 (64%)	0.1
**Apgars >7 at 1**	25/25	23/25	NS
**Apgars >7 at 5**	25/25	25/25	NS
**Birth Weight (grammes)**	3530 (2715–4230)	3537.5 (2830–4250)	0.16
**Gestational Age (weeks) at delivery**	39 (37–41)	38 (35–40)	0.01sq
**Birth Weight Centile**	75 (3–95)	90 (25–100)	0.007
**Placental weight**	588 (452–705)	580 (398–755)	0.9
**Admission to NICU** *	0/25 (0%)	9/25	0.001
**Composite poor outcome (%)** *	0/25 (0%)	7/25 (28%)	0.01

T1DM: Type 1 Diabetes Mellitus. BMI: body mass index. Composite poor outcome included Apgar scores less than 7 at 5 minutes, cord pH less than 7.2 or admission to neonatal intensive care for an indication other than hypoglycaemia.

*Admission to NICU (neonatal intensive care) for any reason. Data for BMI, Birth Weight, Gestational age at delivery, Birth Weight Centile and Placental Weight presented as median (range).

Twelve participants (48%) with T1DM had an early HbA1c less than 7%. Birth weight centile correlated with maternal HbA1c at 14 weeks (r = 0.48, p = 0.016) and at 36 weeks gestation (r = 0.39, p = 0.04).

### Maternal and Fetal Growth Factors (Figure 1)

**Figure 1:**
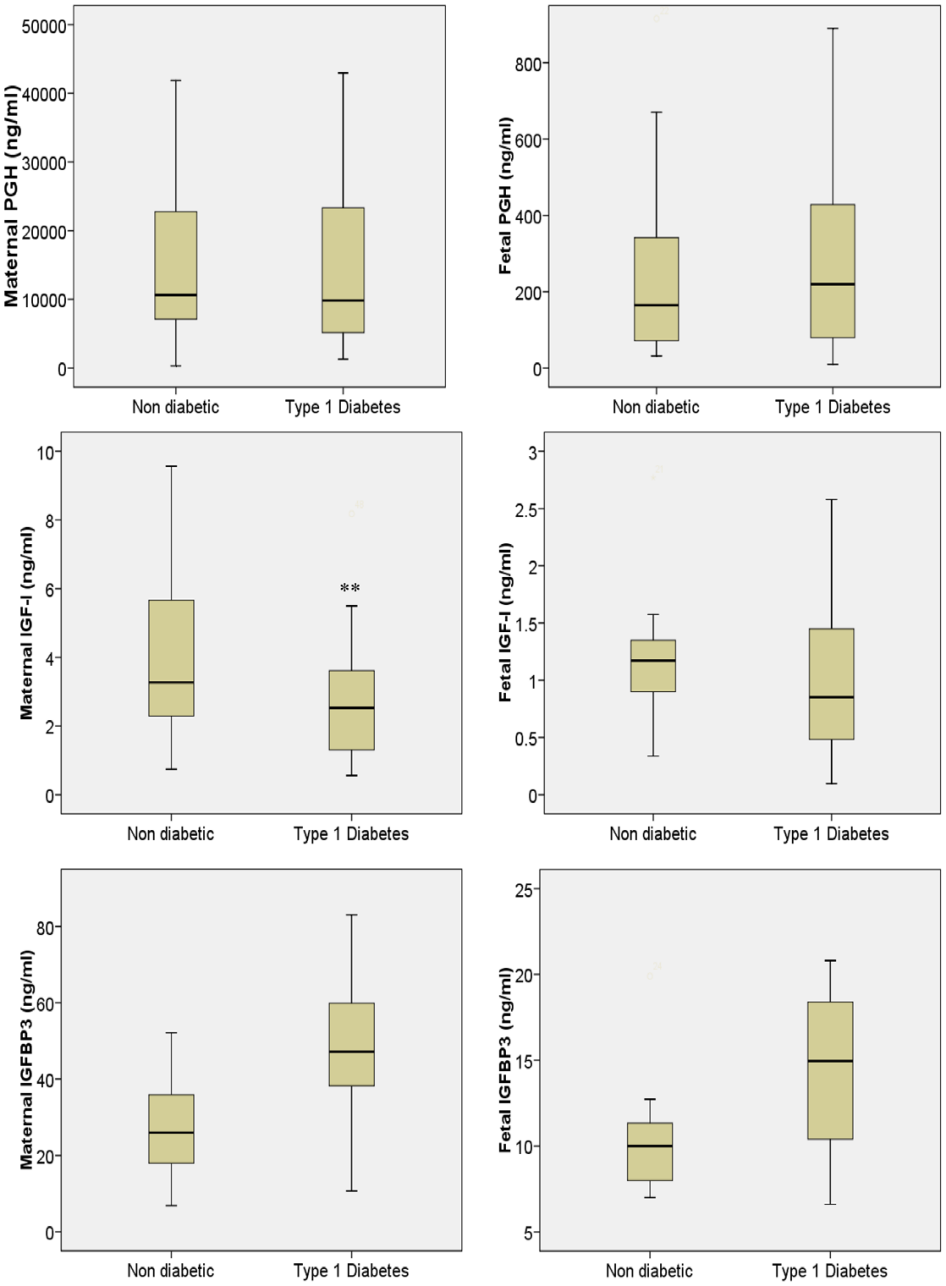
Maternal and Fetal placental growth hormone, IGF-I, and IGFBP3 in non diabetic and Type 1 diabetic pregnancy.

There was no significant difference in maternal or fetal PGH between non-diabetic and T1DM pregnancies, (Table 2). Maternal IGF-I was lower in T1DM mothers compared with non-diabetic controls. There was no difference in fetal IGF-I between infants of diabetic mothers compared with infants of non-diabetic controls. Maternal IGFBP3 was significantly higher in both T1DM mothers compared with non-diabetic controls and fetal serum of infants of diabetic mothers compared with infants of non-diabetic controls.

**Table 2:** Maternal and Fetal placental growth hormone, IGF-I, and IGFBP3 in non diabetic and Type 1 diabetic pregnancy.

	Non-diabetic pregnancy (n = 25)	T1DM pregnancy (n = 25)	p value
**Maternal PGH (ng/ml)**	10639.4 (322.09–89,121.8)	9822.9 (1,293.2–86,679.6)	0.7
**Fetal PGH (ng/ml)**	164.72 (31–1796)	219.9 (10–1208)	0.5
**Maternal IGF-I (ng/ml)**	3.27 (0.74–11.17)	2.26 (0.3–8.18)	0.036
**Fetal IGF-I (ng/ml)**	1.17 (0.31–8.74)	0.76 (0.09–5.33)	0.18
**Maternal IGFBP3(ng/ml)**	25.95 (6.54–52.03)	47.1 (10.71–124.73)	0.001
**Cord IGFBP3 (ng/ml)**	10 (7–19.8)	14.95 (6.61–41.24)	0.001

Data presented as median (range). PGH placental growth hormone, IGF-I insulin like growth factor 1, IGFBP3 insulin like growth factor binding protein 3.

### Correlation between growth factors

In ND and T1DM combined, there was a significant positive correlation between maternal and fetal PGH (r = 0.28, p = 0.04); there was no other significant correlations between any of the other maternal and fetal growth factors. There was no correlation between growth factors in the non-diabetic group alone. In the T1DM group there was a positive correlation between maternal IGF-I and fetal PGH (p = 0.02). In addition, maternal IGFBP3 positively correlated to both maternal PGH (0.61, p = 0.001) and fetal PGH (0.44, p = 0.02).

### Correlation between growth factors and birth weight

In the total group there were no direct correlations between any of the growth factors and birth weight or birth weight centile. Amongst controls alone no significant correlations or differences were found between growth factors and birth weight/birth weight centile. In the diabetic group alone, maternal PGH positively correlated to birth weight (0.42, p = 0.02), and to birth weight centile (r = 0.41, p = 0.03). Of note, fetal PGH did not correlate with birth weight.

### Glycaemia (Diabetic group alone)

Regarding maternal glycaemia (as measured by maternal HbA1c), there was no significant correlation between maternal or cord PGH, IGF-I or IGFBP3 with HbA1c measured in early pregnancy, 14 weeks gestational age (GA), 20 weeks GA or 36 weeks GA.

Comparison was made between levels of PGH, IGF-I and IGFBP3 based on HbA1c more or less than 7% in the three trimesters; fetal IGF-I was higher in women with HbA1c greater than 7% (n = 14) in early pregnancy compared with those with a low early pregnancy HbA1c (n = 11) (1.08 ng/ml (0.5–5.2 ng/ml), vs. 0.4 ng/ml (0.09–1.6 ng/ml) p = 0.01). There was no difference in fetal IGF-I at any other time in pregnancy based on HbA1c>7%.

There was no difference in other growth factors based on HbA1c less than and equal to/more than 7% in first, second or third trimesters. There was no difference in birth weights in women with HbA1c >/<7% in any trimester.

In comparing levels of PGH, IGF-I and IGFBP3 based on HbA1c more or less than median for that trimester, fetal IGF-I was also higher in women with an HbA1c more than median in both early pregnancy and 14 weeks gestational age. (*Early pregnancy* IGF-I in cord blood 0.52 ng/ml (0.09–1.6 ng/ml) in women with HbA1c less than median vs. 1.17 (0.5–5.2 ng/ml) p = 0.01; *14 weeks GA* IGF-I cord 0.61 ng/ml (0.09–1.6 ng/ml) in women with HbA1c less than median vs. 1.18 ng/ml (0.4–5.2 ng/ml) in women with HbA1c more than median at 14 weeks GA, p = 0.04).

There was no further difference in IGFI based on HbA1c </> median at 20 or 36 weeks gestation; there was no difference in growth factor levels based on HbA1c </>median at other gestational ages.

A significant negative correlation was found between maternal IGF-I and length of diabetes (r = −0.621, p = 0.01). There were no other significant correlations between maternal or cord growth factors and length of maternal diabetes.

### Ultrasound markers (Diabetic group only)

Maternal PGH correlated significantly with EFW at both 33 (r = 0.4, p = 0.02) and 36 (r = 0.42,p = 0.03) weeks gestation, but not at 30 weeks gestation (r = 0.3, p = 0.1). There were no other significant correlations between EFW and any other growth factor, nor was there a significant correlation between growth factors and fetal abdominal circumference at any measured time points.

Maternal PGH correlated with fetal AAW at 33 weeks gestation (r = 0.48, p = 0.019) but not at 30 weeks (r = 0.1, p = 0.6) or 36 weeks gestation (r = 0.2, p = 0.4).

Maternal PGH correlated with fetal BPD at 30 weeks (r = 0.61, p = 0.02) and 33 weeks (r = 0.45, p = 0.03). There was no other significant correlation between PGH and other skeletal markers or with other growth factors and markers of skeletal growth. Of note, fetal PGH did not correlate with fetal size or birth weight.

### Clinical characteristics

There was no significant difference between maternal or fetal growth factors regarding mode of delivery, gender of infant or composite poorer perinatal outcome.

### Placental characteristics

Placentae in the diabetic group were the same weight as those of non-diabetic controls (non diabetic 588 (452–705 g) vs. T1DM 580 g (398–755 g), p = 0.9). Placental weight correlated to maternal PGH only within the diabetic group (r = 0.42, p = 0.43), there was no other significant correlation between placental weight and maternal or fetal growth factors in either the non-diabetic nor T1DM groups.

### Western immunoblot analysis

**Figure 2:**
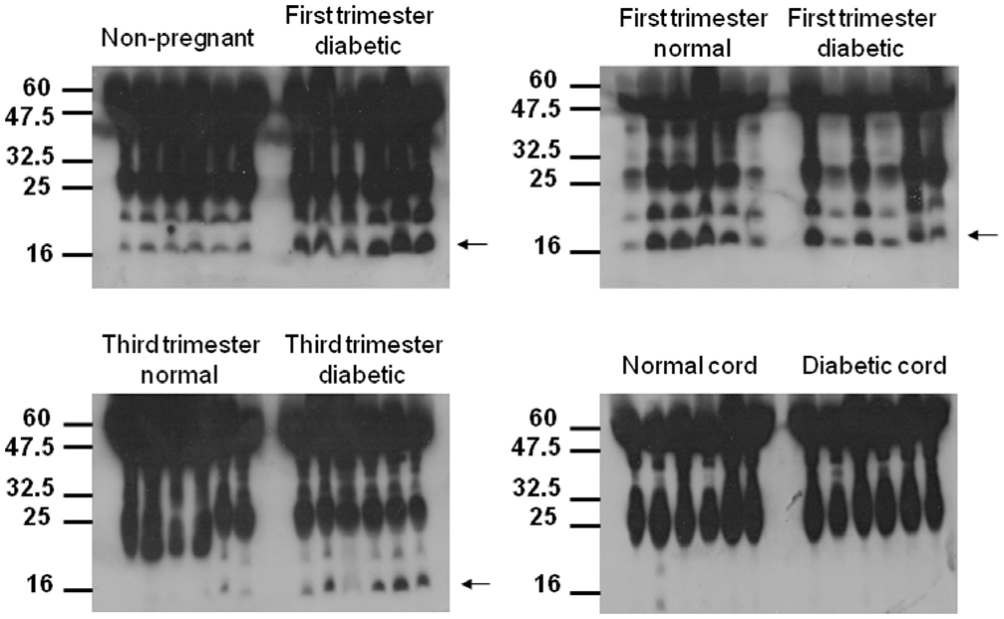
Western immunoblot of IGFBP3 in maternal and fetal serum in non-diabetic (non-pregnant, first trimester, third trimester and cord samples) and type 1 diabetes (first trimester, third trimester and cord samples).

Our ELISA data indicated that IGFBP3 levels were elevated in type 1 diabetic mothers (Fig. 1). IGFBP3 is inactivated by proteolysis in vivo. To determine if changes in IGFBP3 detected by ELISA were accompanied by increased IGFBP3 proteolysis, serum from different patient groups were probed by Western blotting. Comparison of control first versus third trimester indicated that the levels of a 16 kDa proteolytic fragment of IGFBP3 decreased as pregnancy progressed (Fig. 2). Interestingly, levels of this IGFBP3 fragment did not appear to decrease as much in diabetic patients in the third versus first trimester compared to non-diabetic controls. These data suggest that increased proteolysis of IGFBP3 may be a feature of late-stage type diabetic pregnancy.

## Discussion

This study is the first to report detectable PGH in fetal blood of type 1 diabetic mothers. Maternal and fetal PGH levels were found to be similar to those of non-diabetic controls. Although there was no difference in crude birth weight measurements, women with T!DM delivered at an earlier gestational age and the birth weight centile (correcting birth weight for gestational age at delivery) was higher in the T1DM group compared to the non-diabetic group. Although fetal PGH did not correlate with fetal growth, maternal PGH in the diabetic group was significantly correlated with antenatal measurements of fetal adiposity and fetal skeletal growth as well as to birth weight and birth weight centile. IGF-I in maternal diabetic serum was lower than that of normal controls. IGFBP3, a major regulator of IGF, was detected at higher levels in diabetic maternal and fetal blood. Western blot analysis of IGFBP3 suggests that the apparent increase in IGFBP3 levels in mothers with T1DM may be due to increased proteolysis of IGFBP3, though the rise in IGFBP3 in fetal blood appears to be genuine, since there was no apparent difference in fetal IGFBP-3 proteolysis as determined by western immunoblot analysis.

### Placental Growth Hormone in fetal blood

Previously it was thought that PGH was only secreted into the maternal circulation and was not detected in fetal blood [20]. The initial studies investigating placental growth hormone used radioimmunoassay with monoclonal antihuman GH antibodies [20], high levels of pituitary GH were found in the fetal serum but no PGH. Subsequent studies used a sensitive solid phase immuno-radiometric assay whose lowest level of detection was 0.4 ng/ml. Recently a study investigating PGH in pregnancies affected by pre-eclampsia detected fetal PGH for the first time; the authors conclude this was due to the use of an enzyme linked immunosorbent assay with a lower limit of detection of 0.01 ng/ml which would not be affected by th presence of GH binding protein (a potential confounder in earlier studies) [21]. We have confirmed the presence of fetal PGH and at levels similar to those previously reported.

### A role for PGH in the fetus

PGH is secreted from the syncytiotrophoblast in a non-pulsatile fashion [22]. It is traditionally believed that only substances with a molecular weight less than 1 kDa can cross the placental barrier [23]; as PGH has a molecular weight of 22 kDa it seems reasonable to presume that PGH is secreted directly from the syncytiotrophoblast into the fetal circulation. We have found PGH at much lower levels (5%) compared to maternal PGH and therefore its role in fetal growth is debatable. Given that maternal PGH has high somatogenic activity [24], it is also reasonable to presume that fetal PGH may have a similar role in the fetus. We did not, however, find any association between fetal PGH and either ultrasound markers or birth weight. PGH is also thought to have a role in placental development and function [20]. We did not find any association between fetal PGH levels and placental weight or volume, though further more detailed investigations of the functional role of fetal PGH are required to confirm this. Finally, in addition to the PGH gene, a second gene (thought to code for the yet to be defined Growth Hormone Variant 2, GHV1 being PGH) has been described [25] which may represent more than 30% of the transcripts of the placental variants of growth hormone. Therefore the fetal PGH detected may not represent all placental growth hormone variants, the remainder of which may have other, as yet undefined, roles.

### Fetal Placental Growth Hormone in pathological pregnancies

Our hypothesis was that fetal PGH would have an essential role in diabetic fetal growth and would be detected in higher levels than controls. While we have already discussed that the role of fetal PGH in normal and diabetic pregnancies remains to be defined, it is of interest that fetal PGH was similar in T1DM pregnancies compared to controls. The only other study to date detecting PGH in fetal blood investigated pregnancies affected by pre-eclampsia, pre-eclampsia and infants delivered small for gestational age (SGA) and pregnancies with SGA infants alone, both compared to controls. Fetal PGH was significantly higher in infants of women with pre-eclampsia. The researchers postulated that maternal PGH was a compensatory mechanism to preserve fetal growth and failure of this mechanism may result in a SGA infant [21].

### Maternal PGH in diabetic pregnancy

It is interesting that maternal PGH correlated with ultrasound-derived estimates of fetal weight, fetal skeletal growth and with fetal anterior abdominal wall thickness. These are new findings, and suggest that maternal PGH is related to fetal size and not necessarily just to fetal adiposity.

Glucose also has an affect on both PGH and IGF. The absence of glucose has been shown to profoundly increase PGH in vitro [26] as does low concentrations of glucose [27]. This effect of glucose on PGH may be limited to pregnancies affected with diabetes, as PGH has been shown to remain stable in glucose challenge tests in non-diabetic human pregnancies [28]. Despite this association, we did not find a relationship between PGH and HbA1c; also free PGH does not correlate with maternal insulin requirements [29]. Women with severe hypoglycaemia in pregnancy (defined as five or more hypoglycaemic attacks) had 25% lower IGFI in pregnancy compared to women with T1DM and few or no episodes of hypoglycaemia [30]. This finding is of interest in the context of IGF1 being significantly lower in women with a lower HbA1c in the first pregnancy, as we have shown in this study.

Glycaemia itself has a significant effect on fetal weight: the HAPO study showed a relationship between maternal glycaemia and birth weight even in those without defined gestational diabetes, and in diabetic pregnancies maternal glycaemia may explain 6–26% of the variance in birth weight. In this study, we showed that even early HbA1c correlated with birth weight centile, as well as third trimester HbA1c.

The third trimester of pregnancy is a time of relative insulin resistance, when the carbohydrate metabolism of the mother is altered so as to defer fuels to the growing demands of the growing fetus [31]. This insulin resistance is mediated in part by placental hormones, including placental growth hormone, human placental lactogen, leptin and resistin [32]. Therefore as well as glycaemia affecting PGH, it is reasonable to assume that PGH may have an effect on glycaemia. We did not find any association between PGH and maternal HbA1c or fructosamine (results not shown); had we studied maternal insulin requirements or maternal serum glucose different results may have been found but for pragmatic reasons these investigations were not done.

### Insulin like Growth Factor I

IGF production in the liver is stimulated by endogenous insulin. In non-pregnant individuals with diabetes, IGF-I is lower in diabetic serum compared to non-diabetic serum. This may be due to a combination of factors, including hepatic GH resistance, insufficient portal insulinisation, inadequate nutrition and exogenous insulin administration bypassing the liver and thus not stimulating IGF production [33]. We have shown that the length of diabetes negatively correlates with level of maternal IGF-I, suggesting that the differences outlined above become more entrenched with increasing duration of diabetes. We also showed that infants born to mothers with lower IGF-I levels were more likely require admission to the NICU, though this may be confounded by the maternal diabetic effect.

### Insulin Like Growth Factor Binding Protein 3

We have shown that serum IGFBP3 levels increased in both diabetic mothers and their infants. We hypothesized that this may be artifactual due to increased IGFBP3 proteolysis. We investigated this by performing Western immunoblot analysis of IGFBP3. The appearance of a band at 16 kD suggests that there is increased proteolysis in maternal serum in the third trimester. In addition, this proteolysis appears to occur after the first trimester, as no band at 16 kD was seen in the diabetic serum in early pregnancy samples. To our knowledge this is the first time that increased proteolysis of IGFBP3 has been shown in human diabetic pregnancies. If the primary function of IGFBP3 is to bind IGF-I and reduce its bioavailability, then increased proteolysis in the third trimester may increase the level of IGF-I, perhaps in order to stimulate growth during the period of maximum fetal growth.

This band does not appear in fetal diabetic serum, suggesting that the increase in fetal IGFBP3 diabetic mothers is a genuine increase in total IGFBP3 rather than increased IGFBP3 fragments. This is supported by other studies showing an increased fetal IGFBP3 in diabetic pregnancy [34]. The higher IGFBP3 may thus increase binding of IGF-I and reduce its bioavailability. This may well be a component of the overall mechanism suppressing IGF-I activity in fetal blood discussed above.

One limitation of the study was the number of participants involved; as with any study a larger number may have changed the results. Secondly, only PGH, IGFI and IGFBP3 were measured. PGH and Pituitary GH share a common binding protein, GHBP, which regulates the concentration of free –biologically active - PGH in serum. GHBP has been shown to decrease with advancing gestational age in T1DM, with a corresponding increase in free PGH [11]. Free PGH increases in maternal serum in T1DM at 28 and 36 weeks gestation, with both total PGH and free PGH correlating to fetal ponderal index (and thus adiposity). Free PGH, IGFBP3, IGFBP1, IGF2 and GHBP may explain 40% of the variance in birth weight. Therefore the exclusion of GHBP is a limitation of this study. For pragmatic reasons we chose to study PGH, IGF1 and IGFBP3 considering these the most relevant in third trimester. In retrospect it may have been useful to assess total PGH and GHBP as well as free PGH in fetal serum to see if these measurements would have correlated to fetal growth.

These results suggest that in T1DM pregnancies, the maternal and fetal PGH-IGF-I-IGFBP3 is altered, providing further information why pregnancies of diabetic women are at increased risk of fetal macrosomia.

## Methods

### Patient cohort

This was a prospective cohort study of patients attending the hospital between 2005 and 2008. With institutional ethics approval (National Maternity Hospital, Dublin, Ireland) and informed written consent, women underwent routine antenatal care, but in addition maternal blood was drawn at 36 weeks gestation. Following delivery of the baby but before delivery of the placenta, cord blood was obtained from the umbilical vein. Blood was centrifuged and plasma stored at −20°C.

For the purpose of ease of sample collection the majority of cord samples were obtained from women undergoing elective caesarean section (CS), with the indication for delivery being either breech presentation or elective repeat delivery in both groups. Women with T1DM underwent earlier delivery than non-diabetic pregnancies. The baseline CS rate in our unit is 18%.

Type 1 Diabetes was defined as a metabolic disease characterized by hyperglycaemia as a result of insulin deficiency. None of the diabetic group had any medical problems other than diabetes. Women who screened negative for gestational diabetes were invited to participate as controls. In the Republic of Ireland screening for gestational diabetes is based on risk factor assessment rather than universal screening. Risk factors for gestational diabetes include a family history in a first-degree relative of diabetes, previous gestational diabetes, previous unexplained macrosomia, persistent glycosuria, unexplained polyhydramnious or maternal obesity, amongst others. Should a patient have any of these risk factors, she underwent a glucose tolerance test. All pregnancies resulted in the delivery of a single live infant.

### Clinical data collection

Clinical data on both normal and T1DM pregnancies was obtained prospectively from the labour ward database, ultrasound department database and patient records. Participant demography, delivery details and infant outcomes such as birth weight, birth weight centile and Apgar scores were recorded. Poor perinatal outcome was defined as any of the following: Apgar at 1 or 5 minutes less than 7, cord pH less than 7.2 or admission to the NICU for indication other than hypoglycaemia. In addition measurements of glycaemic control (HbA1c) at early pregnancy (first visit), second trimester (14 and 20 weeks gestation) and third trimester (36 weeks gestation) were recorded. The median gestational age at first visit was 5 weeks (range 4–7 weeks). Gestation was defined clinically as number of weeks since date of certain last menstrual period or by ultrasound assessment if dates were uncertain. Birth weight was weight following delivery as measured by a paediatrician or midwife using standardised scales (Seca 1 Model 335, Vogel and Halke, Germany). The gestation and gender appropriate centile was determined using the growth charts (Child Growth Foundation, London 1996) used in this institution.

### Ultrasound measurements

Women with T1DM underwent the routine third trimester ultrasound assessment for fetal wellbeing and fetal growth at 30, 33 and 36 weeks' gestation. At each examination fetal growth (as measured by bi-parietal diameter, head circumference, abdominal circumference and femur length), measurement of fetal anterior abdominal wall thickness [5] fetal wellbeing (bio-physical profile) and Doppler assessment (of the umbilical artery and middle cerebral artery) was performed. Ultrasound measurements were performed by one of three operators (MH, NR, FMcA). All examinations were performed transabdominally using either a Toshiba Xario (Toshiba Medical Systems Corporation, Japan) or Voluson 730 Expert, (GE Medical Systems, Germany) equipped with curved array transducers. Growth factors were correlated with ultrasound markers including estimated fetal weight (EFW), abdominal circumference (AC), and fetal anterior abdominal wall thickness (AAW) (as measurements of fetal adiposity) as well as femur length (FL) bi-parietal diameter (BPD) and head circumference (HC) (markers of fetal skeletal growth).

### Statistics

Statistical analysis was performed using SPSS version 16. Data was assessed for normality using Shapiro Wilk and p-p plot; normally distributed data was compared using t test (mean +/− standard error). Proportions were compared using chi^2^ test or Fishers test as appropriate. P values<0.05 were considered significant.

### Experiment

#### ELISA analysis

Placental Growth Hormone was measured by enzyme-linked immunoassay (Diagnostic Systems Laboratories, Texas) with an intra-assay precision of 6.7% and inter-assay precision of 6.4%. The minimal detectable level of hPGH is 8 pg/ml in this assay. Samples reporting the maximum detectable levels were repeated at diluted levels (×2, ×10, ×20) until measurements were within the linear range were recorded. Insulin Like Growth Hormone I was measured by enzyme-linked immunoassay (R & D Systems, Minneapolis) with an intra-assay precision of 4.3% and inter-assay precision of 7.5%. The minimal detectable level of IGF-I is 0.026 ng/ml in this assay. Insulin Like Growth Factor Binding Protein 3 (IGFBP3) was measured by enzyme-linked immunoassay (R & D Systems, Minneapolis) with an intra-assay precision of 4.1% and inter-assay precision of 6.9%. The minimal detectable level of IGFBP3 is 0.05 ng/ml in this assay. Samples were analysed on a Molecular Devices multiwell plate reader at 450 nm. Readings were normalised using a standard curve and concentrations extrapolated and analysed.

#### Western Blot

For the western blot analysis, seven groups were analysed: non pregnant non diabetic controls, first trimester non-diabetic controls, first trimester T1DM, third trimester non-diabetic controls, third trimester T1DM and cord samples from infants of the non-diabetic and T1DM third trimester participants. Fetal and maternal serum samples were obtained from the same consenting participants as previously described. 50 microlitres of serum from five patients per indicated group were separated by 10% (w/v) SDS-PAGE and transferred to PVDF membrane using standard techniques. Membranes were blocked with 3% (w/v) milk solution and incubated overnight with IGFBP3 primary antibody (1∶1000 dilution, Santa Cruz, sc-6004). Membranes were washed with TBS-Tween and incubcated with 1∶10,000 anti-rabbit HRP for 60 min. Membranes were incubated with ECL reagents and immunoreactive bands visualised with X-ray film.
