# Correction: Maternal and Fetal Placental Growth Hormone and IGF Axis in Type 1 Diabetic Pregnancy

**DOI:** 10.1371/annotation/b3c08210-a703-4986-aa97-c8513c5aff5f

**Published:** 2012-05-30

**Authors:** Mary F. Higgins, Noirin E. Russell, Paul A. Crossey, Kristine C. Nyhan, Derek P. Brazil, Fionnuala M. McAuliffe

The following information was missing from the Funding section: Derek Brazil and Kristine Nyhan were funded by Science Foundation Ireland as part of the UCD Diabetes Research Centre. 

